# CD47 and FOXP3^+^ Regulatory Immunity in Colorectal Cancer: A Conceptual Framework for Coordinated Immunosuppression

**DOI:** 10.3390/cancers18162614

**Published:** 2026-08-13

**Authors:** Qijie Li, Anello Marcello Poma, Donghao Tang, Paola Vignali, Rossella Bruno, Elisabetta Macerola, Beatrice Fuochi, Clara Ugolini

**Affiliations:** 1 Department of Surgical, Medical, Molecular Pathology and Critical Area, University of Pisa, 56126 Pisa, Italy; qijie.li@phd.unipi.it (Q.L.); marcello.poma@med.unipi.it (A.M.P.); b.fuochi@studenti.unipi.it (B.F.); 2 Department of Translational Research and New Technologies in Medicine and Surgery, University of Pisa, 56126 Pisa, Italy; d.tang1@studenti.unipi.it (D.T.); paola.vignali@med.unipi.it (P.V.); 3 Unit of Pathological Anatomy 3, University Hospital of Pisa, 56126 Pisa, Italy; r.bruno@ao-pisa.toscana.it (R.B.); elisabetta.macerola@for.unipi.it (E.M.)

**Keywords:** colorectal cancer, CD47, FOXP3, regulatory T cells, tumor microenvironment

## Abstract

In colorectal cancer (CRC), tumor progression is influenced by immunosuppressive tumor microenvironment (TME), in which innate immunity and adaptive immunity play an important role. CD47 is one of the key molecules in the process. It sends a “don’t eat me” signal to macrophages by binding to signal regulatory protein alpha (SIRPα), thus helping tumor cells escape immune clearance. Forkhead box P3 (FOXP3)^+^ regulatory immune cells further suppress antitumor T-cell responses. Here, based on a review of the literature and publicly available transcriptomic data, we propose that CD47 expression and FOXP3^+^ regulatory T cells in CRC are interconnected components of a broader myeloid–regulatory immunosuppressive phenotype, rather than a simple linear CD47–FOXP3 pathway. Evidence from cancer studies and exploratory GEPIA3/TIMER3.0 analyses supports a weak and method-dependent association between CD47 expression, FOXP3 transcripts, and estimated Treg infiltration. Hippo/Yes-associated protein/transcriptional coactivator with PDZ-binding motif (YAP/TAZ) signaling serves as a potential upstream program contributing to this immune context.

## Simple Summary

Colorectal cancer can grow and spread partly because cancer cells learn how to avoid immune attack. One important signal is CD47, which helps tumor cells escape clearance by immune cells called macrophages. Another important part of this process involves FOXP3-positive regulatory immune cells, which can weaken anti-tumor immune responses. In this review, we discuss whether these two immune-suppressive features may work together in colorectal cancer. Rather than suggesting a simple direct pathway between CD47 and FOXP3, we propose that they may reflect a broader immune environment in which both innate and adaptive anti-tumor responses are reduced. Public database analyses showed only weak and method-dependent associations, which do not establish a direct relationship between CD47 and FOXP3-related regulatory immunity. This framework may help guide future studies of immune escape and the prospective evaluation of combined therapeutic strategies targeting myeloid immune checkpoints and regulatory immune suppression.

## 1. Introduction

Colorectal cancer (CRC) remains a major challenge in oncology. Globally, it ranks third in cancer incidence and second in cancer-related mortality, with more than 1.9 million new cases and approximately 0.9 million deaths reported annually [1,2]. CRC progression depends on interactions between tumor cells and the tumor microenvironment (TME) [3]. In CRC, tumor cells interact continuously with stromal cells, macrophages, lymphocytes, and other immune populations, and these interactions can shape immune escape and treatment response [4]. Notably, immune evasion is recognized as a fundamental hallmark of cancer, and as precancerous colorectal lesions evolve into CRC, the surrounding microenvironment adapts to support tumour growth and promote immune evasion [3].

CD47 is one of the best-known innate immune checkpoints in cancer [5]. Through its interaction with signal regulatory protein alpha (SIRPα) on myeloid cells, CD47 limits phagocytosis and allows tumor cells to avoid macrophage-mediated clearance [6,7]. CD47 is frequently overexpressed in a wide range of malignancies, including hematologic and solid tumors, and its elevated expression is associated with poor prognosis, highlighting its clinical relevance in cancer progression [8]. Although the CD47–SIRPα axis has attracted considerable therapeutic interest, its immunological significance in CRC extends beyond macrophage evasion.

Regulatory T cells (Tregs) are key immunoregulatory components of the TME that suppress anti-tumor immune responses, partly through inhibition of cytotoxic CD8^+^ T-cell activity [9]. In CRC, discordant results have been reported concerning the prognostic value of intratumor Treg infiltration [10]. Studies found that the number of intratumoral forkhead box P3 (FOXP3)^+^ cells was positively associated with lymph node metastases [11]. Treg-derived interleukin-10 (IL-10) has been shown to promote tumor progression and metastasis in CRC [12]. Importantly, CD47 overexpression and FOXP3^+^ immune-cell infiltration are both observed in CRC, but these features may reflect the same immunosuppressive context rather than two independent events [10,13,14,15]. This review therefore examines whether CD47-mediated innate immune escape and FOXP3^+^ regulatory T cells in CRC may represent interconnected components of a broader myeloid–regulatory immunosuppressive phenotype, rather than two independent events. This focus extends our previous review of CD47 in CRC by specifically examining its potential coordination with FOXP3-associated regulatory immunity.

## 2. Evidence

### 2.1. CD47–Treg Immunoregulatory Interactions Across Biological Contexts

CD47 also serves as an immunoregulatory receptor on T lymphocytes, beyond its role as a tumor-cell “don’t-eat-me” signal [16]. Engagement of CD47 by thrombospondin-1 (TSP-1) restrains TCR-driven T-cell activation through mechanisms involving reduced H_2_S-dependent extracellular signal-regulated kinase phosphorylation and diminished induction of activation markers such as CD69, CD25, and interleukin-2 [17,18,19]. Thus, CD47 is not only a macrophage checkpoint; it also has a direct role in T-cell activation control and immune homeostasis [20,21].

CD47 is also linked to Treg biology. Almost all CD4^+^CD25^+^FOXP3^+^ regulatory Tregs exhibit CD47 expression, and increased CD47 levels have been reported in peripheral Tregs during inflammatory states [22]. CD47–TSP-1 signaling is involved in promoting the conversion of CD4^+^CD25^−^ T cells into Tregs, and CD47 also plays a role in maintaining the homeostasis of activated CD103^+^Foxp3^+^ Treg populations [23,24]. CD47 deficiency does not eliminate Treg suppressive function, but it changes Treg subset balance and proliferation. This places CD47 closer to Treg maintenance than to suppression itself [24].

These findings suggest that CD47 influences T-cell fate and immune resolution [25,26,27]. The SIRPα-binding form is selectively retained in a subset of proliferating cells that favor memory formation, whereas loss of this form is associated with terminal effector differentiation and contraction [25,28]. CD47 deficiency also alters the contraction phase of antigen-specific CD4^+^ T cells, indicating a role in T-cell persistence [28]. In parallel, the CD47/SIRPα axis has been implicated in T-cell–mediated antitumor responses and in the regulation of myeloid activity within the anticancer immunity cycle [26,29].

### 2.2. CD47–Treg Associations Across Multiple Cancer Types

Across tumor types, CD47 expression has been associated with FOXP3^+^ Treg-related immunosuppression. High CD47 expression has been reported to correlate with increased FOXP3^+^ Treg infiltration and reduced effector T-cell dominance [30,31]. Tumor-infiltrating Tregs can co-express CD47 and CTLA-4, which indicates that CD47 expression extends beyond tumor cells and characterizes suppressive regulatory immune compartments [31].

Through engaging SIRPα on myeloid cells, CD47 delivers a “don’t eat me” signal that suppresses macrophage-mediated phagocytosis, thereby facilitating tumor escape from innate immune surveillance [32]. CD47 is broadly overexpressed in gastrointestinal tumors, including gastric and colorectal cancers, and its elevated expression is commonly linked to poor clinical outcomes [14]. Within these tumors, CD47 overexpression is observed in immunosuppressive TME. In Epstein–Barr virus-associated gastric carcinoma, higher CD47 expression is associated with worse survival and a lower CD8^+^/FOXP3^+^ ratio, suggesting reduced effector dominance [33,34]. Similar associations have been reported in non-small-cell lung cancer, where tumor CD47 expression correlates with FOXP3^+^ tumor-infiltrating lymphocytes [35].

A similar pattern appears in hematologic malignancies. In adult T-cell leukemia/lymphoma, CD47-positive cases show a significantly higher frequency of FoxP3 expression than CD47-negative cases [36]. In ovarian cancer and other solid tumors, elevated CD47 expression correlates with T-cell exhaustion, reduced CD8^+^/FOXP3^+^ ratios, and increased FOXP3^+^ regulatory T-cell infiltration [37,38,39]. Transcriptomic and single-cell analyses also suggest that CD47-high tumors may be enriched for both M2-like macrophages and Tregs [33,38]. Across these tumor types, CD47-high status appears more closely tied to a suppressive immune context than to a direct CD47–Treg pathway [26,40].

### 2.3. CRC–Specific Observations

CD47 is expressed in CRC and can be further induced under therapeutic stress [41]. In rectal cancer patients with poor responses to neoadjuvant radiotherapy, post-irradiation CD47 levels were significantly elevated compared with pre-treatment biopsies, suggesting that radiotherapy can induce CD47 upregulation in vivo. Functionally, the CD47–SIRPα axis helps cancer cells evade innate immune surveillance [42,43]. Although macrophages can phagocytose tumor cells, malignant cells express CD47 as a “don’t eat me” signal to inhibit this process [43,44]. In CRC, radiotherapy can upregulate both CD47 and PD-L1, and radiotherapy-induced CD47 upregulation has been observed in multiple human solid tumors [41].

Independent clinical and experimental studies also support a role for CD47 in CRC immune evasion [40]. CD47 is overexpressed in colon adenocarcinoma tissues and has been associated with advanced tumor stage, lymphovascular invasion, poor differentiation, distant metastasis, impaired disease-free survival, and reduced overall survival [14,39,45]. Increased tumor-infiltrating FOXP3^+^ T cells have been documented in CRC specimens, though their prognostic value differs from other solid tumors [10,46,47,48]. Functional studies further show that CD47 blockade enhances CD8^+^ T-cell–mediated antitumor activity in colon cancer models [40]. These observations suggest that CD47 upregulation and FOXP3^+^ immune infiltration can coexist in CRC.

Although FOXP3^+^ cell density is often used as a marker of immunoregulatory activity, FOXP3 expression alone does not identify functionally suppressive human Tregs [49,50,51]. Human FOXP3^+^CD4^+^ T cells are functionally heterogeneous and may include both suppressive and non-suppressive populations [52,53,54]. Therefore, FOXP3^+^ T-cell populations are not considered synonymous with functionally suppressive CD4^+^FOXP3^+^ effector Tregs, and FOXP3^+^ cell infiltration in CRC is better viewed as a marker of regulatory immune activity rather than proof of uniformly suppressive Treg accumulation. Tumors enriched for highly suppressive effector Treg populations may differ clinically from those containing higher proportions of non-suppressive FOXP3^+^ T-cell populations [55].

### 2.4. Mechanistic Hints from Other Cancers

#### 2.4.1. CD47 Links Innate Immune Escape to Adaptive T-Cell Suppression

To evaluate whether CD47 directly regulates tumor cell growth, CD47 was silenced in NK/T-cell lymphoma cells using shRNA transfection [56]. Stable CD47-knockdown cells showed no significant changes in proliferation, apoptosis, or cell-cycle progression, but were more susceptible to M2 macrophage-mediated phagocytosis. Similarly, treatment with anti-CD47 antibodies enhanced macrophage-mediated tumor-cell clearance. CD47 primarily functions in immune evasion rather than directly driving tumor cell growth.

CD47 blockade can also promote adaptive antitumor immunity. This effect relies on STING-dependent type I interferon signaling and dendritic cell–mediated cross-priming of CD8^+^ T cells [57]. The therapeutic effects of CD47 blockade are lost in T-cell–deficient mice, indicating that tumor regression depends on adaptive T-cell activation as well as enhanced phagocytosis. CD47 inhibition initiates a coordinated innate-to-adaptive immune response.

CD47–SIRPα blockade enhances dendritic-cell cross-presentation and promotes CD8^+^ T-cell–mediated cytotoxicity in colon cancer models [57,58]. When combined with PD-1/PD-L1 blockade, CD47-targeted therapy produces a stronger increase in CD8^+^ T-cell cytotoxic activity, highlighting its role in linking phagocytic processes with adaptive immunity [40,59]. In addition, CD47-targeted approaches can remodel the TME by reducing intratumoral FOXP3^+^ Tregs while increasing CD8^+^ T-cell infiltration and effector activation [60,61]. By increasing tumor-cell uptake and antigen cross-presentation, it can convert phagocytosis into a CD8^+^ T-cell response [58].

#### 2.4.2. CD47 Expression Is Regulated by Oncogenic and Therapeutic Stress Pathways

CD47 upregulation in tumors occurs within broader oncogenic and stress-response programs. Multiple oncogenic and stress-related transcription factors regulate CD47 expression. NF-κB, Myc, and HIF-1 directly bind to regulatory regions of the CD47 gene and drive its transcription in response to inflammatory signaling and hypoxia [27,62,63,64]. In addition to transcriptional induction, CD47 can modulate innate immune sensing pathways, including cGAS–STING signaling, thereby influencing antigen cross-presentation and T-cell priming [57,65]. CD47 upregulation is therefore not an isolated surface-marker change. It is part of stress-responsive transcriptional programs driven by hypoxia, Myc activity, and inflammatory signaling.

Therapeutic stress represents another upstream driver of CD47 expression. In CRC, irradiation-induced DNA damage activates the ATR-mediated double-strand break repair pathway, resulting in upregulation of both CD47 and PD-L1 [41]. These molecules subsequently interact with SIRPα and PD-1, respectively, to suppress phagocytosis and antigen cross-presentation by antigen-presenting cells. Combined blockade of CD47/SIRPα and PD-1 after radiotherapy reduced FoxP3^+^ Treg infiltration, increased the CD8^+^/Treg ratio, and was accompanied by decreases in MDSCs, M2-like macrophages, and monocytes.

#### 2.4.3. CD47 May Cooperate with Myeloid and Regulatory Immune Circuits

High CD47 expression or specific CD47 polymorphisms have been associated with poor survival and increased metastasis in several cancers [7,66]. In CRC, CD47 overexpression frequently coexists with CD68^+^/CD206^+^ M2 tumor-associated macrophages (TAMs) [67]. M2 macrophages secrete IL-10 and IL-8 to promote immunosuppressive microenvironments that favor regulatory T-cell dominance. This connects CD47 upregulation to TAM-rich, immunoregulatory niches within the TME [39]. In gastric cancer peritoneal metastases, Galectin-3 has been shown to cooperate with CD47 to suppress phagocytosis and T-cell immunity [32].

Tregs are recruited into tumors through chemokine gradients, including CCL2, CCL22, and CXCL12, as well as macrophage-derived signals [68,69]. This recruitment establishes an immune tolerance microenvironment that supports tumor persistence [70]. A plausible route from CD47 to Treg enrichment is through macrophages. CD47 protects tumor cells from macrophage clearance, while TAM-rich regions can produce cytokines and chemokines that attract or stabilize Tregs. In this model, CD47 does not need to recruit Tregs directly; it helps maintain a myeloid environment that favors Tregs. Engagement of CD47 by TSP-1 promotes the differentiation of CD25^+^FOXP3^+^ Tregs from human CD4^+^ T cells [23,71]. These induced Tregs exhibit suppressive activity, while disruption of TSP-1 signaling enhances effector T-cell activation and cytokine production. This effect occurs outside tumor contexts and shows that CD47 signaling can bias CD4^+^ T-cell fate toward a regulatory phenotype. Thus, CD47 signal may promote adaptive immune tolerance by affecting regulatory T-cell differentiation [72].

In tumor models, the antitumor efficacy of a CTLA-4 × SIRPα bispecific antibody depends on CD47 expression on Treg cells [31]. This treatment selectively depletes intratumoral Tregs through Fc-dependent effector mechanisms while largely sparing peripheral Tregs. These findings indicate that Treg-intrinsic CD47 can affect the persistence or therapeutic targeting of Tregs. Under steady-state conditions, genetic ablation of CD47 leads to a gradual and selective expansion of activated CD103^+^Foxp3^+^ Tregs with age [24]. This suggests that CD47 also helps control the homeostasis of specific Treg subsets. Additionally, TSP-1–CD47 signaling can suppress T-cell activation and has been implicated in T-cell exhaustion under chronic stimulation, so the CD47 axis may affect not only Tregs but also the effector T cells they suppress [26,73,74,75,76].

These observations suggest a myeloid-mediated connection between CD47 and FOXP3^+^ regulatory T cells. In this model, CD47 does not need to recruit Tregs directly. It helps preserve a macrophage-rich, weakly antigen-presenting niche in which regulatory T-cell programs are favored.

## 3. Hypothesis

### 3.1. Working Hypothesis

Across tumor types, high CD47 expression is often associated with regulatory T-cell enrichment or reduced CD8^+^/FOXP3^+^ ratios, suggesting a CD47-high immunosuppressive phenotype (see Section 2.2). In CRC, both CD47 expression and FOXP3^+^ regulatory immune infiltration have been linked to immune modulation and disease progression, but a direct CD47–FOXP3 pathway has not been established (see Section 2.3). We therefore hypothesize that CD47-high CRC represents a broader myeloid–regulatory immune state. In this proposed state, CD47 expression on tumor cells limits macrophage-mediated phagocytosis and may consequently reduce tumor-antigen release, dendritic-cell activation, and CD8^+^ T-cell priming. In parallel, tumor-associated macrophages and tumor-derived chemokines may promote the recruitment, maintenance, or functional stabilization of FOXP3^+^ regulatory T-cell populations. Hippo pathway dysregulation and YAP/TAZ activation are considered a possible upstream program that coordinates CD47 upregulation, myeloid remodeling, and Treg-enriched immunity [77]. In this conceptual framework, FOXP3^+^ regulatory T-cell populations are regarded as indicators of a regulatory immune context rather than definitive evidence of functionally suppressive Tregs.

### 3.2. Hippo/YAP–TAZ Signaling as a Potential Upstream Regulator

Hippo/YAP–TAZ signaling may be an important upstream pathway connecting CD47 up-regulation with the formation of immunosuppressive microenvironment. YAP/TAZ activation can reshape TME by inducing the expression of immune escape-related molecules and regulating chemokines that affect immune cell recruitment (Section 2.4). Although it has not been clearly reported in CRC that YAP/TAZ can directly regulate CD47 transcription, direct regulation of CD47 by YAP/TAZ and a direct mechanistic link between YAP/TAZ activation and FOXP3^+^ regulatory T-cell enrichment have not yet been demonstrated in CRC. Therefore, Hippo/YAP–TAZ signaling is presented as a potential upstream coordinating program rather than an established mechanism.

In addition to its significance as a “don’t-eat-me” signal in tumor cells, CD47 signal is also involved in the maintenance of T-cell homeostasis and regulatory T-cell balance. In non-tumor settings, altered CD47 signal can affect the activated CD103^+^Foxp3^+^ Treg population and weaken the antigen-specific T-cell response, suggesting that there is a functional relationship between CD47 and Treg biology [24]. We speculate that YAP/TAZ activation may contribute to an immune context in which CD47-related immune escape and Treg-associated immunosuppression coexist.

### 3.3. A Conceptual Model of Cooperative Immune Suppression

**Figure 1:**
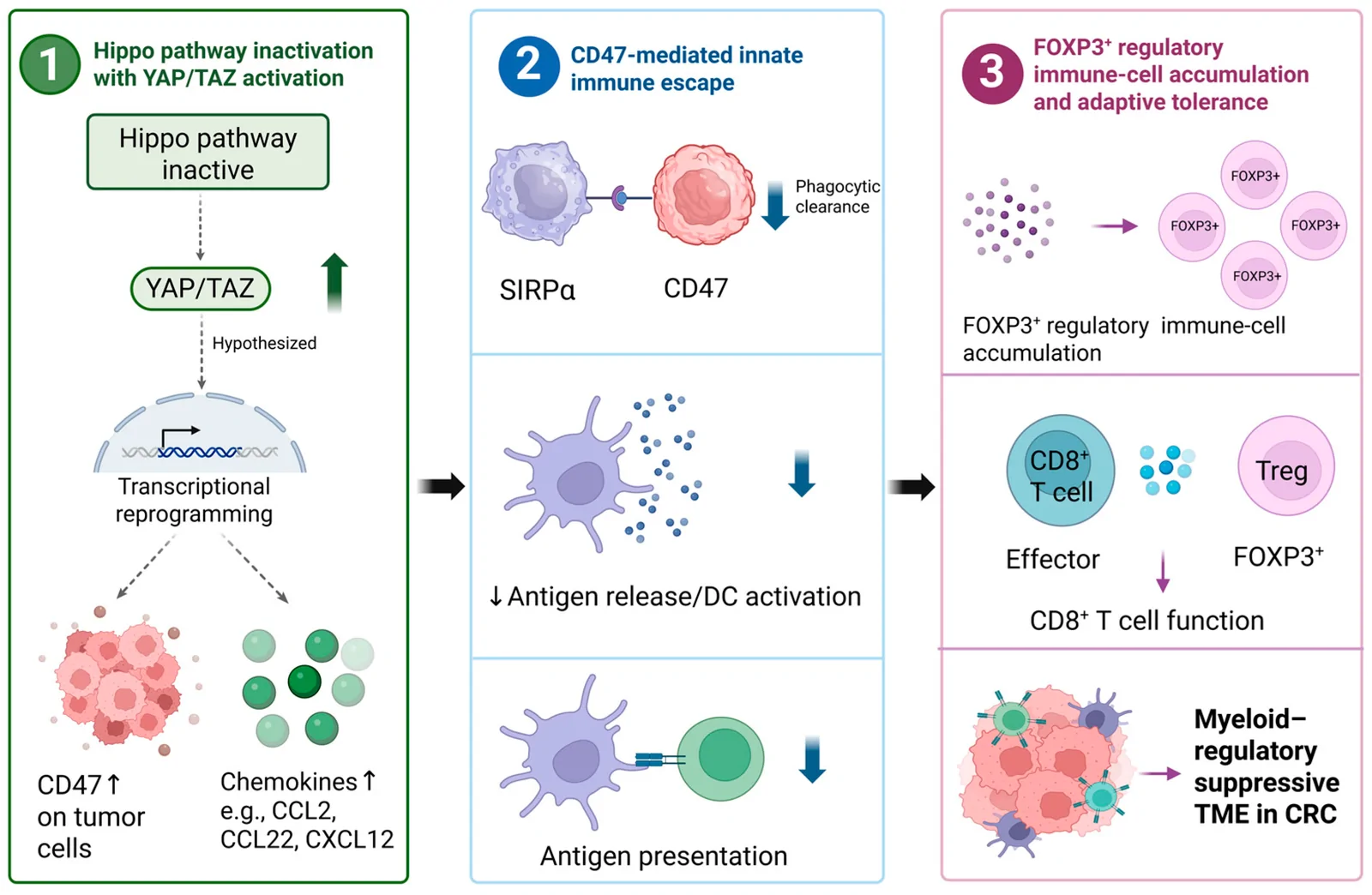
A conceptual model of CD47-associated myeloid–regulatory immune suppression in CRC. Upward and downward arrows indicate proposed increases or decreases in the indicated molecular, cellular, or functional features, respectively. Hippo pathway inactivation with YAP/TAZ activation is proposed to promote CD47 upregulation and chemokine induction, favoring CD47-mediated innate immune escape with FOXP3^+^ regulatory immune-cell accumulation and reduced CD8^+^ T-cell function. Solid black arrows indicate proposed conceptual links between the three modules. Dashed arrows indicate hypothesized relationships that have not been established in CRC.

We speculate that Hippo pathway inactivation and YAP/TAZ activation may enhance immune evasion characteristics in CRC cells, as summarized in Figure 1. In this process, the up-regulation of CD47 may help tumor cells escape macrophage-mediated clearance, thereby reducing antigen release and weakening dendritic cell activation, antigen presentation and downstream CD8^+^ T-cell activation. In parallel, YAP/TAZ-associated chemokine programs and TAM-derived signals may also provide support for the recruitment or stability of FOXP3^+^ regulatory immune cells. The result is a myeloid–regulatory suppressive TME in which innate clearance and adaptive effector immunity are restrained together.

### 3.4. Predicted Immunological Consequences

If this model is correct, CD47-high/YAP–TAZ-active CRC should show several immune features. First, enhanced CD47 expression would be expected to impair macrophage-mediated phagocytosis and dendritic-cell activation, limiting antigen presentation and antitumor immune initiation. Second, recruitment or functional stabilization of FOXP3^+^ Tregs would reinforce a Treg-dominated immune landscape. In such a setting, effector CD8^+^ T-cell activity may be suppressed, reducing cytotoxic function and tumor control. Third, impaired innate immune clearance and reinforced adaptive immune tolerance would favor a self-sustaining immunosuppressive microenvironment. This would appear as a low effector-to-regulatory T-cell ratio, weak inflammatory signaling, and stronger immune escape.

## 4. Public Database Analysis

To explore the proposed CD47–Treg framework in CRC, we analyzed public transcriptomic data using GEPIA3 and TIMER3.0. GEPIA3 correlation analyses were performed using Pearson’s correlation coefficient in TCGA COAD (*n* = 290), READ (*n* = 93), and the combined CRC cohort (*n* = 383), based on log_2_(TPM + 1)-transformed expression data. TIMER3.0 was used to assess tumor-purity-adjusted Spearman correlations between CD47 expression and estimated Treg infiltration in COAD and READ. A nominal *p* < 0.05 was used to identify statistical significance. Because these analyses were exploratory, *p* values were not adjusted for multiple comparisons and were interpreted cautiously. Both databases were accessed on 27 May 2026.

### 4.1. Correlation Between CD47 and FOXP3 Expression

**Figure 2:**
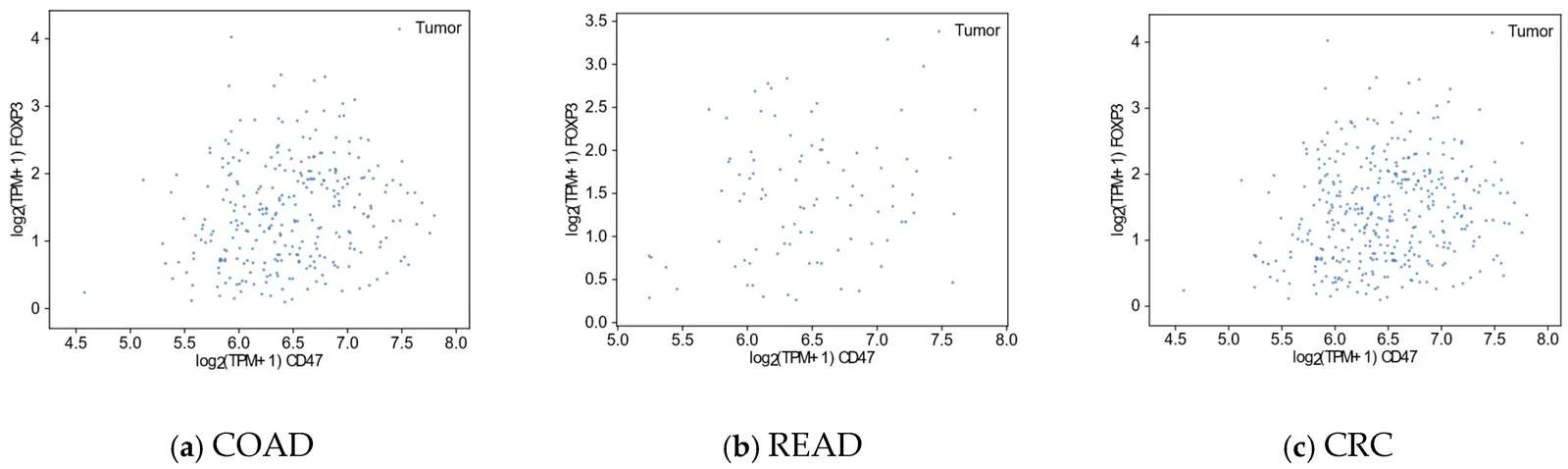
GEPIA3 analysis of the correlation between CD47 and FOXP3 expression in CRC. (**a**) COAD. (**b**) READ. (**c**) CRC, including COAD and READ samples. Correlation coefficients, *p* values, and tumor sample numbers are shown in each panel.

In COAD, CD47 expression showed a weak but statistically significant positive correlation with FOXP3 expression (*r* = 0.157, *p* = 0.00756, *n* = 290). In READ, CD47 expression also showed a weak positive correlation with FOXP3, although this association did not reach statistical significance (*r* = 0.179, *p* = 0.0864, *n* = 93). When COAD and READ samples were combined as a CRC cohort, CD47 and FOXP3 showed a weak but statistically significant positive correlation (*r* = 0.162, *p* = 0.00151, *n* = 383). The weak correlation does not support a simple CD47–FOXP3 linear relationship. Because GEPIA3 is based on bulk transcriptomic data, the cellular source of CD47 expression cannot be resolved. The GEPIA3 correlation results are summarized in Table 1 and Figure 2.

### 4.2. Association Between CD47 Expression and Estimated Treg Infiltration

**Figure 3:**
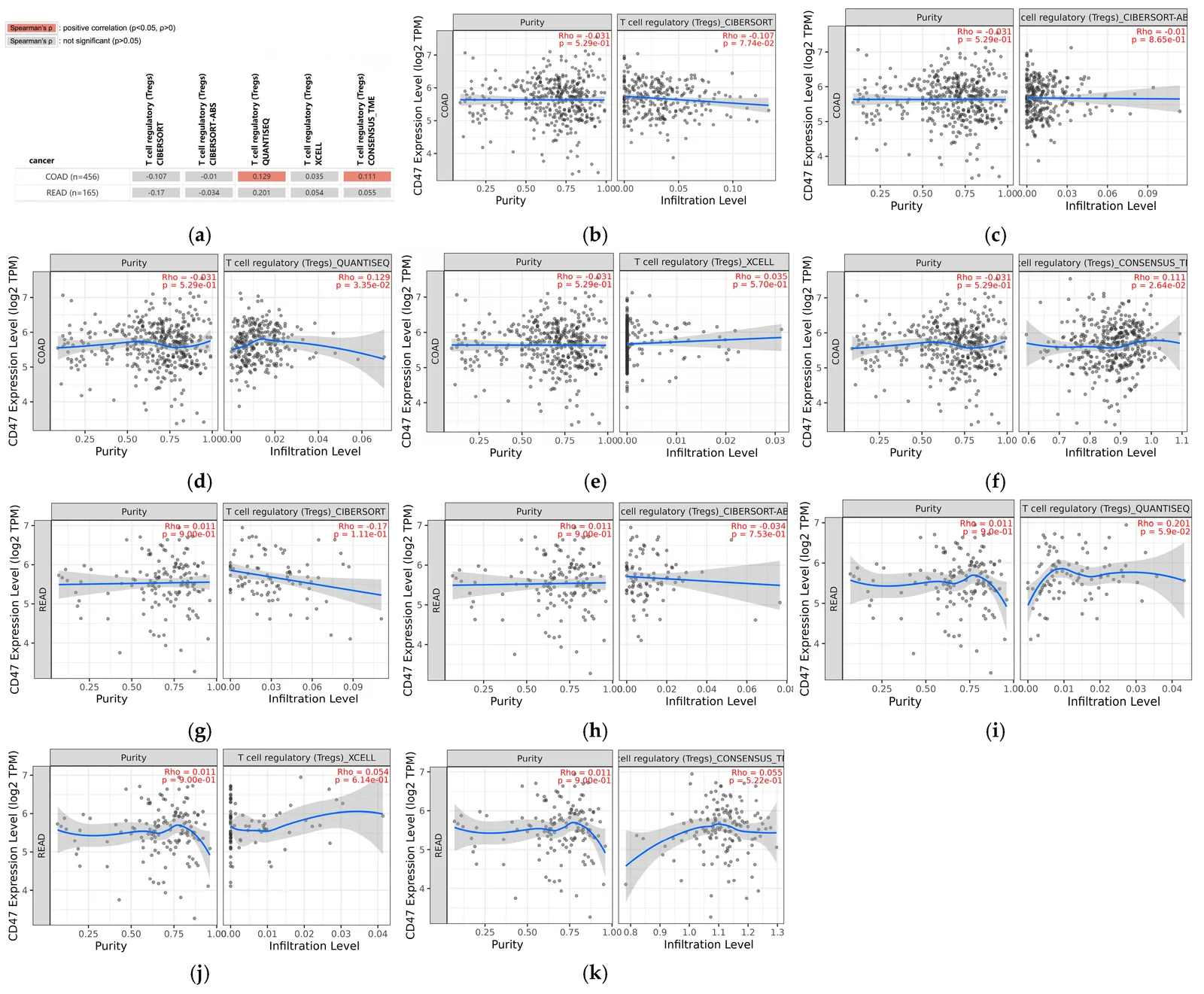
Association between CD47 expression and estimated Treg infiltration in CRC using TIMER3.0. (**a**) Heatmap summarizing correlations across five Treg infiltration estimates methods in COAD and READ. (**b**–**f**) Dot plots for COAD using CIBERSORT, CIBERSORT-ABS, quanTIseq, xCell, and ConsensusTME. (**g**–**k**) Dot plots for READ using CIBERSORT, CIBERSORT-ABS, quanTIseq, xCell, and ConsensusTME. Spearman correlation coefficients and *p* values after adjustment for tumor purity are shown in each panel.

We next assessed the association between CD47 expression and estimated Treg infiltration using TIMER3.0 [78]. In COAD, CD47 expression showed weak positive correlations with estimated Treg infiltration using quanTIseq (Rho = 0.129, *p* = 0.0335) and ConsensusTME (Rho = 0.111, *p* = 0.0264), whereas no statistically significant associations were observed using CIBERSORT, CIBERSORT-ABS, or xCell. In READ, none of the five methods showed a statistically significant association between CD47 expression and estimated Treg infiltration. The associations between CD47 expression and estimated Treg infiltration were weak and inconsistent across tumor cohorts and infiltration estimation methods. The complete TIMER3.0 results are summarized in Figure 3.

The database analyses identified only weak and method-dependent associations between CD47 expression, FOXP3 transcripts, and estimated Treg infiltration. TIMER3.0 results were inconsistent across infiltration estimation methods, with some methods showing weak positive associations and others showing null or weak negative associations. Therefore, these exploratory findings should be regarded as hypothesis-generating and are insufficient to validate the proposed biological framework.

## 5. Therapeutic Implications and Perspectives

Within the proposed myeloid–regulatory framework, CD47 blockade may restore myeloid-mediated tumor clearance, whereas Treg-targeted approaches may relieve adaptive immune suppression; their combination may therefore address both components of the proposed immunosuppressive state.

### 5.1. CD47 Blockade Beyond Phagocytosis

CD47 blockade has traditionally been considered to increase macrophage-mediated phagocytosis by blocking the CD47–SIRPα “don’t eat me” signal [37,57,79]. Other findings showed that the immunological effects of CD47 inhibition are not restricted to innate phagocytic clearance [57]. In immunocompetent tumor model, CD47 blockade promotes dendritic cell-mediated tumor antigen-specific T cells, linking innate phagocytic response with adaptive antitumor immunity. CD8^+^ T-cell depletion reduces the therapeutic effect of anti-CD47 therapy, which demonstrates that CD8^+^ T-cell responses are critical for tumor control. CD47 blockade starts with macrophage phagocytosis, but can extend into dendritic-cell activation and CD8^+^ T-cell immunity.

### 5.2. Potential Synergy with Treg-Targeted Approaches

In the proposed CD47–Treg framework, the therapeutic rationale is complementary rather than redundant. The combined targeting of CTLA-4 and CD47 on Treg cells can preferentially eliminate the immunosuppressive Tregs in tumors and enhance the anti-tumor immune response in various solid tumor models, including MC38 and CT26 colon cancer models [31]. CD47 inhibition can promote macrophage activation and tumor cell phagocytosis, thus improving antigen presentation and CD8^+^ T-cell activation. Treg-targeted therapy is helpful to reduce suppressive constraints in TME [80,81]. A humanized anti-CTLA-4 × SIRPα heterodimer preferentially targets CTLA-4-high tumor-infiltrating Tregs while reducing CD47-associated immune evasion, thereby minimizing the impact on peripheral cells with low CTLA-4 expression [31]. In preclinical tumor models, this dual targeting strategy can selectively reduce the Treg level in tumors and enhance anti-tumor immunity.

In MC38 and CT26 CRC models, CTLA-4 blockade combined with cordycepin treatment also showed similar immunomodulatory effects, which could increase CD8^+^ T-cell infiltration and decrease FOXP3^+^ Treg abundance [82]. These preclinical findings provide a biological rationale for further testing combined myeloid-checkpoint and Treg-directed strategies in CRC.

### 5.3. Challenges and Considerations

While CD47 targeted therapy has shown anti-tumor potential in preclinical models, evidence supporting combined CD47- and Treg-targeted treatment in CRC remains preclinical [31]. Evidence from other solid-tumor models provides additional but indirect support. Clinical evidence relevant to CRC includes a phase 1b/2 study in which magrolimab combined with cetuximab showed early evidence of tolerability and potential antitumor activity in heavily pretreated patients with advanced CRC [83]. This regimen did not involve Treg-targeted treatment or FOXP3-based patient selection. Because CD47 is expressed on the surface of many normal cells, systemic CD47 blockade may cause on-target toxicity and narrow its therapeutic window [84]. Furthermore, CD47 blockade affects not only tumor cells and macrophages, but also dendritic cells, effector T cells and Tregs. Therefore, when evaluating CD47-related therapeutic strategies, it is necessary to consider both its anti-tumor effects and its impact on the overall TME. Therapeutic responses will probably depend on the immune context around CD47-high cells.

The proposed CD47–Treg relationship in CRC remains incompletely defined. Public database analyses suggest only weak and method-dependent associations between CD47 expression and estimated Treg infiltration and do not support a strong direct CD47–FOXP3 transcript-level correlation. CD47-high/FOXP3-high CRC has not yet been clinically validated as a therapeutic subgroup. Key priorities are to test whether CD47-high CRC tumors contain suppressive, not merely FOXP3-positive, Treg populations, and whether CD47 blockade changes their recruitment, stability, or function. Future studies should evaluate whether CD47-high CRCs with myeloid-cell enrichment and functionally suppressive Treg features are particularly responsive to combined CD47- and Treg-targeted strategies.

## 6. Discussion

The available evidence does not point to a simple CD47–FOXP3 relationship in CRC. CD47-high tumors likely belong to a broader immune-suppressive context in which macrophage clearance is limited, antigen presentation is weakened, and FOXP3^+^ regulatory immune cells are more prone to accumulate. This interpretation is compatible with the weak CD47–FOXP3 correlations observed in GEPIA3 and the method-dependent Treg infiltration signals from TIMER3.0. These exploratory analyses did not account for mismatch-repair/microsatellite-instability status, tumor location, molecular subtype, stage, or treatment exposure, all of which may influence CD47 expression, FOXP3 transcription, and immune-cell composition. These unmeasured factors may therefore contribute to the weak and heterogeneous associations observed.

In CRC, this distinction is critical because FOXP3^+^ infiltration does not equate to uniformly suppressive Treg accumulation. Human FOXP3^+^CD4^+^ T cells are heterogeneous, and CRC tumors can harbor both suppressive effector Tregs and non-suppressive FOXP3^+^ T-cell populations [10,85]. The key issue is whether CD47-high CRC tissues are enriched for suppressive Treg programs, such as FOXP3^+^CTLA-4^+^, CD25^+^, CCR4^+^, or IL-10-producing regulatory subsets. A myeloid-mediated explanation remains the most reasonable: CD47 can protect tumor cells from macrophage-mediated clearance, while TAM-rich regions may provide cytokines and chemokines that support regulatory immune infiltration [26,37,86]. Thus, Treg enrichment would reflect a CD47-associated myeloid environment with weak phagocytic activity, impaired antigen presentation, and increased regulatory immune pressure.

Hippo/YAP–TAZ signaling may provide an upstream route into this state [87]. Direct evidence that YAP/TAZ controls CD47 or FOXP3 in CRC remains limited, but this pathway is relevant because it can influence immune-evasion molecules, inflammatory signaling, chemokine expression, and myeloid remodeling. Therapeutic and cellular-therapy studies further support a broader role for CD47. In localized prostate cancer, androgen deprivation therapy altered tumor-cell CD47 expression together with T-cell infiltration, FOXP3^+^ Treg expansion, and antigen-presentation programs [71], while adoptive T-cell studies show that CD47–SIRPα signaling protects transferred T cells from macrophage-mediated clearance and improves CAR-T persistence [88,89].

From a therapeutic perspective, CD47 expression alone is insufficient to determine suitability for CD47-targeted therapy. It is more reasonable to combine the up-regulation of CD47 with TME characteristics, including myeloid cell enrichment, inhibitory Treg-related markers, low CD8^+^/FOXP3^+^ ratio and impaired antigen-presenting function. Future research should test whether the high expression region of CD47 in CRC overlaps with M2-like macrophages, inhibitory Tregs and exhausted CD8^+^ T cells in spatial distribution, and explore whether CD47/SIRPα blockade, macrophage reprogramming or YAP/TAZ regulation can restore antigen-presenting function and enhance CD8^+^ T-cell-mediated tumor control.

## 7. Conclusions

CD47-mediated myeloid immune evasion and FOXP3^+^ T-cell heterogeneity are established features of CRC. However, the weak and method-dependent database associations do not establish a direct CD47–FOXP3 pathway. The proposed model should be tested by multiplexed imaging and functional assays.

## Figures and Tables

**Table 1:** GEPIA3 correlation analysis of CD47 and FOXP3 expression in TCGA CRC cohorts.

TCGA Tumor	Gene Set A	Gene Set B	*r*	*p*-Value	*n*_Tumor
COAD	CD47	FOXP3	0.157	0.00756	290
READ	CD47	FOXP3	0.179	0.0864	93
CRC	CD47	FOXP3	0.162	0.00151	383

## Data Availability

No new datasets were generated in this study. All analyzed data are publicly available from GEPIA3: https://gepia3.bioinfoliu.com/ (accessed on 27 May 2026) and TIMER3.0: https://compbio.cn/timer3/ (accessed on 27 May 2026).

## Abbreviations

The following abbreviations are used in this manuscript:

COAD	Colon adenocarcinoma
CRC	Colorectal cancer
CTLA-4	Cytotoxic T-lymphocyte-associated protein 4
FOXP3	Forkhead box P3
GEPIA3	Gene Expression Profiling Interactive Analysis 3
HIF-1	Hypoxia-inducible factor 1
IL-10	Interleukin-10
MDSCs	Myeloid-derived suppressor cells
NF-κB	Nuclear factor kappa B
PD-1	Programmed cell death protein 1
PD-L1	Programmed death-ligand 1
READ	Rectum adenocarcinoma
SIRPα	Signal regulatory protein alpha
STING	Stimulator of interferon genes
TAMs	Tumor-associated macrophages
TCR	T-cell receptor
TME	Tumor microenvironment
Tregs	Regulatory T cells
TSP-1	Thrombospondin-1
YAP/TAZ	Yes-associated protein/transcriptional coactivator with PDZ-binding motif

## References

[1] Sung H., Ferlay J., Siegel R.L., Laversanne M., Soerjomataram I., Jemal A., Bray F. (2021). Global Cancer Statistics 2020: GLOBOCAN Estimates of Incidence and Mortality Worldwide for 36 Cancers in 185 Countries. CA Cancer J. Clin..

[2] Roshandel G., Ghasemi-Kebria F., Malekzadeh R. (2024). Colorectal Cancer: Epidemiology, Risk Factors, and Prevention. Cancers.

[3] Kennel K.B., Greten F.R. (2025). The immune microenvironment of colorectal cancer. Nat. Rev. Cancer.

[4] Hanahan D. (2022). Hallmarks of Cancer: New Dimensions. Cancer Discov..

[5] Li Q., Vignali P., Tang D., Martinelli G., Fuochi B., Sparavelli R., Poma A.M., Bruno R., Macerola E., Ugolini C. (2025). Expression and Clinical Significance of CD47 in Colorectal Cancer: A Review. Cancers.

[6] Veillette A., Chen J. (2018). SIRPα-CD47 Immune Checkpoint Blockade in Anticancer Therapy. Trends Immunol..

[7] Fujiwara-Tani R., Sasaki T., Ohmori H., Luo Y., Goto K., Nishiguchi Y., Mori S., Nakashima C., Mori T., Miyagawa Y. (2019). Concurrent Expression of CD47 and CD44 in Colorectal Cancer Promotes Malignancy. Pathobiology.

[8] Ahvati H., Roudi R., Sobhani N., Safari F. (2025). CD47 as a potent target in cancer immunotherapy: A review. Biochim. Biophys. Acta Rev. Cancer.

[9] Usman A.N., Ahmad M., Sinrang A.W., Natsir S., Takko A.B., Ariyandy A., Ilhamuddin I., Eragradini A.R., Hasan I.I., Hasyim S. (2023). FOXP3 regulatory T cells on prognosis of breast cancer. Breast Dis..

[10] Zhuo C., Xu Y., Ying M., Li Q., Huang L., Li D., Cai S., Li B. (2015). FOXP3+ Tregs: Heterogeneous phenotypes and conflicting impacts on survival outcomes in patients with colorectal cancer. Immunol. Res..

[11] Suzuki H., Chikazawa N., Tasaka T., Wada J., Yamasaki A., Kitaura Y., Sozaki M., Tanaka M., Onishi H., Morisaki T. (2010). Intratumoral CD8(+) T/FOXP3 (+) cell ratio is a predictive marker for survival in patients with colorectal cancer. Cancer Immunol. Immunother..

[12] Shiri A.M., Fard-Aghaie M., Bedke T., Papazoglou E.D., Sabihi M., Zazara D.E., Zhang S., Lücke J., Seeger P., Evers M. (2024). Foxp3 + Treg-derived IL-10 promotes colorectal cancer-derived lung metastasis. Sci. Rep..

[13] Stebbing J., Bullock A. (2025). CD47 as a potential predictive biomarker in colorectal cancer. J. Immunother. Cancer.

[14] Otuagomah J., Newcomb A., Gwag T., Wang S. (2025). CD47: An immunoregulatory nexus in liver and gastrointestinal disorders. eGastroenterology.

[15] Kim M., Grimmig T., Grimm M., Lazariotou M., Meier E., Rosenwald A., Tsaur I., Blaheta R., Heemann U., Germer C.T. (2013). Expression of Foxp3 in colorectal cancer but not in Treg cells correlates with disease progression in patients with colorectal cancer. PLoS ONE.

[16] Li Z., He L., Wilson K., Roberts D. (2001). Thrombospondin-1 inhibits TCR-mediated T lymphocyte early activation. J. Immunol..

[17] Miller T.W., Kaur S., Ivins-O’Keefe K., Roberts D.D. (2013). Thrombospondin-1 is a CD47-dependent endogenous inhibitor of hydrogen sulfide signaling in T cell activation. Matrix Biol..

[18] Ridnour L.A., Isenberg J.S., Espey M.G., Thomas D.D., Roberts D.D., Wink D.A. (2005). Nitric oxide regulates angiogenesis through a functional switch involving thrombospondin-1. Proc. Natl. Acad. Sci. USA.

[19] Kaur S., Schwartz A.L., Miller T.W., Roberts D.D. (2015). CD47-dependent regulation of H2S biosynthesis and signaling in T cells. Methods in Enzymology.

[20] Miller T.W., Wang E.A., Gould S., Stein E.V., Kaur S., Lim L., Amarnath S., Fowler D.H., Roberts D.D. (2012). Hydrogen sulfide is an endogenous potentiator of T cell activation. J. Biol. Chem..

[21] Autio A., Wang H., Velázquez F., Newton G., Parkos C.A., Engel P., Engelbertsen D., Lichtman A.H., Luscinskas F.W. (2022). SIRPα—CD47 axis regulates dendritic cell-T cell interactions and TCR activation during T cell priming in spleen. PLoS ONE.

[22] Lee N., Shin J.U., Jin S., Yun K.N., Kim J.Y., Park C.O., Kim S.H., Noh J.Y., Lee K.H. (2016). Upregulation of CD47 in Regulatory T Cells in Atopic Dermatitis. Yonsei Med. J..

[23] Grimbert P., Bouguermouh S., Baba N., Nakajima T., Allakhverdi Z., Braun D., Saito H., Rubio M., Delespesse G., Sarfati M. (2006). Thrombospondin/CD47 interaction: A pathway to generate regulatory T cells from human CD4+ CD25- T cells in response to inflammation. J. Immunol..

[24] Van V.Q., Darwiche J., Raymond M., Lesage S., Bouguermouh S., Rubio M., Sarfati M. (2008). Cutting edge: CD47 controls the in vivo proliferation and homeostasis of peripheral CD4+CD25+Foxp3+ regulatory T cells that express CD103. J. Immunol..

[25] Van V.Q., Raymond M., Baba N., Rubio M., Wakahara K., Susin S.A., Sarfati M. (2012). CD47(high) expression on CD4 effectors identifies functional long-lived memory T cell progenitors. J. Immunol..

[26] van Duijn A., Van der Burg S.H., Scheeren F.A. (2022). CD47/SIRPα axis: Bridging innate and adaptive immunity. J. Immunother. Cancer.

[27] Zhang H., Lu H., Xiang L., Bullen J.W., Zhang C., Samanta D., Gilkes D.M., He J., Semenza G.L. (2015). HIF-1 regulates CD47 expression in breast cancer cells to promote evasion of phagocytosis and maintenance of cancer stem cells. Proc. Natl. Acad. Sci. USA.

[28] Van V.Q., Baba N., Rubio M., Wakahara K., Panzini B., Richard C., Soucy G., Franchimont D., Fortin G., Torres A.C. (2012). CD47low status on CD4 effectors is necessary for the contraction/resolution of the immune response in humans and mice. PLoS ONE.

[29] He X., Xu C. (2020). Immune checkpoint signaling and cancer immunotherapy. Cell Res..

[30] Chen Y., Klingen T.A., Aas H., Wik E., Akslen L.A. (2023). CD47 and CD68 expression in breast cancer is associated with tumor-infiltrating lymphocytes, blood vessel invasion, detection mode, and prognosis. J. Pathol. Clin. Res..

[31] Zhang A., Ren Z., Tseng K.F., Liu X., Li H., Lu C., Cai Y., Minna J.D., Fu Y.X. (2021). Dual targeting of CTLA-4 and CD47 on Treg cells promotes immunity against solid tumors. Sci. Transl. Med..

[32] Fan Y., Song S., Li Y., Dhar S.S., Jin J., Yoshimura K., Yao X., Wang R., Scott A.W., Pizzi M.P. (2023). Galectin-3 Cooperates with CD47 to Suppress Phagocytosis and T-cell Immunity in Gastric Cancer Peritoneal Metastases. Cancer Res..

[33] Abe H., Saito R., Ichimura T., Iwasaki A., Yamazawa S., Shinozaki-Ushiku A., Morikawa T., Ushiku T., Yamashita H., Seto Y. (2018). CD47 expression in Epstein-Barr virus-associated gastric carcinoma: Coexistence with tumor immunity lowering the ratio of CD8+/Foxp3+ T cells. Virchows Arch..

[34] Paluskievicz C.M., Cao X., Abdi R., Zheng P., Liu Y., Bromberg J.S. (2019). T Regulatory Cells and Priming the Suppressive Tumor Microenvironment. Front. Immunol..

[35] Giatromanolaki A., Mitrakas A., Anestopoulos I., Kontosis A., Koukourakis I.M., Pappa A., Panayiotidis M.I., Koukourakis M.I. (2022). Expression of CD47 and SIRPα Macrophage Immune-Checkpoint Pathway in Non-Small-Cell Lung Cancer. Cancers.

[36] Yanagida E., Miyoshi H., Takeuchi M., Yoshida N., Nakashima K., Yamada K., Umeno T., Shimasaki Y., Furuta T., Seto M. (2020). Clinicopathological analysis of immunohistochemical expression of CD47 and SIRPα in adult T-cell leukemia/lymphoma. Hematol. Oncol..

[37] Bess S.N., Igoe M.J., Muldoon T.J. (2025). The Physiological and Therapeutic Role of CD47 in Macrophage Function and Cancer. Immunol. Investig..

[38] Yu L., Ding Y., Wan T., Deng T., Huang H., Liu J. (2021). Significance of CD47 and Its Association With Tumor Immune Microenvironment Heterogeneity in Ovarian Cancer. Front. Immunol..

[39] Tzatzarakis E., Hissa B., Reissfelder C., Schölch S. (2019). The overall potential of CD47 in cancer immunotherapy: With a focus on gastrointestinal tumors. Expert. Rev. Anticancer. Ther..

[40] Jia X., Yan B., Tian X., Liu Q., Jin J., Shi J., Hou Y. (2021). CD47/SIRPα pathway mediates cancer immune escape and immunotherapy. Int. J. Biol. Sci..

[41] Hsieh R.C., Krishnan S., Wu R.C., Boda A.R., Liu A., Winkler M., Hsu W.H., Lin S.H., Hung M.C., Chan L.C. (2022). ATR-mediated CD47 and PD-L1 up-regulation restricts radiotherapy-induced immune priming and abscopal responses in colorectal cancer. Sci. Immunol..

[42] Luo X., Shen Y., Huang W., Bao Y., Mo J., Yao L., Yuan L. (2023). Blocking CD47-SIRPα Signal Axis as Promising Immunotherapy in Ovarian Cancer. Cancer Control.

[43] Chen Y., Zhu X., Liu H., Wang C., Chen Y., Wang H., Fang Y., Wu X., Xu Y., Li C. (2023). The application of HER2 and CD47 CAR-macrophage in ovarian cancer. J. Transl. Med..

[44] Gordon S., Martinez F.O. (2010). Alternative activation of macrophages: Mechanism and functions. Immunity.

[45] Tian Q.S., Zhang C., Bao Z.J., Pei Z. (2024). The role of CD47 in immune escape of colon cancer and its correlation with heterogeneity of tumor immune microenvironment. PeerJ.

[46] Ladoire S., Martin F., Ghiringhelli F. (2011). Prognostic role of FOXP3+ regulatory T cells infiltrating human carcinomas: The paradox of colorectal cancer. Cancer Immunol. Immunother..

[47] Salama P., Phillips M., Grieu F., Morris M., Zeps N., Joseph D., Platell C., Iacopetta B. (2009). Tumor-infiltrating FOXP3+ T regulatory cells show strong prognostic significance in colorectal cancer. J. Clin. Oncol..

[48] Xu P., Fan W., Zhang Z., Wang J., Wang P., Li Y., Yu M. (2017). The Clinicopathological and Prognostic Implications of FoxP3+ Regulatory T Cells in Patients with Colorectal Cancer: A Meta-Analysis. Front. Physiol..

[49] Gootjes C., Zwaginga J.J., Roep B.O., Nikolic T. (2024). Defining Human Regulatory T Cells beyond FOXP3: The Need to Combine Phenotype with Function. Cells.

[50] Sakaguchi S. (2005). Naturally arising Foxp3-expressing CD25+CD4+ regulatory T cells in immunological tolerance to self and non-self. Nat. Immunol..

[51] Josefowicz S.Z., Lu L.F., Rudensky A.Y. (2012). Regulatory T cells: Mechanisms of differentiation and function. Annu. Rev. Immunol..

[52] Miyara M., Yoshioka Y., Kitoh A., Shima T., Wing K., Niwa A., Parizot C., Taflin C., Heike T., Valeyre D. (2009). Functional delineation and differentiation dynamics of human CD4+ T cells expressing the FoxP3 transcription factor. Immunity.

[53] Sakaguchi S., Miyara M., Costantino C.M., Hafler D.A. (2010). FOXP3+ regulatory T cells in the human immune system. Nat. Rev. Immunol..

[54] Sugiyama D., Nishikawa H., Maeda Y., Nishioka M., Tanemura A., Katayama I., Ezoe S., Kanakura Y., Sato E., Fukumori Y. (2013). Anti-CCR4 mAb selectively depletes effector-type FoxP3+CD4+ regulatory T cells, evoking antitumor immune responses in humans. Proc. Natl. Acad. Sci. USA.

[55] Saito T., Nishikawa H., Wada H., Nagano Y., Sugiyama D., Atarashi K., Maeda Y., Hamaguchi M., Ohkura N., Sato E. (2016). Two FOXP3+CD4+ T cell subpopulations distinctly control the prognosis of colorectal cancers. Nat. Med..

[56] Qin L., Li Y., Zeng R., He Y., Chen X., Xiao L., Zhou H. (2024). A novel anti-CD47 antibody with therapeutic potential for NK/T-cell lymphoma. Hum. Vaccines Immunother..

[57] Liu X., Pu Y., Cron K., Deng L., Kline J., Frazier W.A., Xu H., Peng H., Fu Y.X., Xu M.M. (2015). CD47 blockade triggers T cell-mediated destruction of immunogenic tumors. Nat. Med..

[58] Tseng D., Volkmer J.P., Willingham S.B., Contreras-Trujillo H., Fathman J.W., Fernhoff N.B., Seita J., Inlay M.A., Weiskopf K., Miyanishi M. (2013). Anti-CD47 antibody-mediated phagocytosis of cancer by macrophages primes an effective antitumor T-cell response. Proc. Natl. Acad. Sci. USA.

[59] Liu X., Liu L., Ren Z., Yang K., Xu H., Luan Y., Fu K., Guo J., Peng H., Zhu M. (2018). Dual Targeting of Innate and Adaptive Checkpoints on Tumor Cells Limits Immune Evasion. Cell Rep..

[60] Wu L., Yu G.T., Deng W.W., Mao L., Yang L.L., Ma S.R., Bu L.L., Kulkarni A.B., Zhang W.F., Zhang L. (2018). Anti-CD47 treatment enhances anti-tumor T-cell immunity and improves immunosuppressive environment in head and neck squamous cell carcinoma. Oncoimmunology.

[61] Lozano T., Conde E., Martín-Otal C., Navarro F., Lasarte-Cia A., Nasrallah R., Alignani D., Gorraiz M., Sarobe P., Romero J.P. (2022). TCR-induced FOXP3 expression by CD8+ T cells impairs their anti-tumor activity. Cancer Lett..

[62] Feng M., Jiang W., Kim B.Y.S., Zhang C.C., Fu Y.X., Weissman I.L. (2019). Phagocytosis checkpoints as new targets for cancer immunotherapy. Nat. Rev. Cancer.

[63] Casey S.C., Tong L., Li Y., Do R., Walz S., Fitzgerald K.N., Gouw A.M., Baylot V., Gütgemann I., Eilers M. (2016). MYC regulates the antitumor immune response through CD47 and PD-L1. Science.

[64] Chang W.T., Huang A.M. (2004). Alpha-Pal/NRF-1 regulates the promoter of the human integrin-associated protein/CD47 gene. J. Biol. Chem..

[65] Chen Q., Sun L., Chen Z.J. (2016). Regulation and function of the cGAS-STING pathway of cytosolic DNA sensing. Nat. Immunol..

[66] Lascorz J., Bevier M., Schönfels W.V., Kalthoff H., Aselmann H., Beckmann J., Egberts J., Buch S., Becker T., Schreiber S. (2013). Association study identifying polymorphisms in CD47 and other extracellular matrix pathway genes as putative prognostic markers for colorectal cancer. Int. J. Colorectal Dis..

[67] Zhang Y., Sime W., Juhas M., Sjölander A. (2013). Crosstalk between colon cancer cells and macrophages via inflammatory mediators and CD47 promotes tumour cell migration. Eur. J. Cancer.

[68] Ireland D.J., Kissick H.T., Beilharz M.W. (2012). The Role of Regulatory T Cells in Mesothelioma. Cancer Microenviron..

[69] McFadden C., Morgan R., Rahangdale S., Green D., Yamasaki H., Center D., Cruikshank W. (2007). Preferential migration of T regulatory cells induced by IL-16. J. Immunol..

[70] Miselis N.R., Lau B.W., Wu Z., Kane A.B. (2010). Kinetics of host cell recruitment during dissemination of diffuse malignant peritoneal mesothelioma. Cancer Microenviron..

[71] Dallos M.C., Obradovic A.Z., McCann P., Chowdhury N., Pratapa A., Aggen D.H., Gaffney C., Autio K.A., Virk R.K., De Marzo A.M. (2024). Androgen Deprivation Therapy Drives a Distinct Immune Phenotype in Localized Prostate Cancer. Clin. Cancer Res..

[72] Sarfati M., Fortin G., Raymond M., Susin S. (2008). CD47 in the immune response: Role of thrombospondin and SIRP-alpha reverse signaling. Curr. Drug Targets.

[73] Avice M.N., Rubio M., Sergerie M., Delespesse G., Sarfati M. (2001). Role of CD47 in the induction of human naive T cell anergy. J. Immunol..

[74] Sato-Hashimoto M., Saito Y., Ohnishi H., Iwamura H., Kanazawa Y., Kaneko T., Kusakari S., Kotani T., Mori M., Murata Y. (2011). Signal regulatory protein α regulates the homeostasis of T lymphocytes in the spleen. J. Immunol..

[75] Seiffert M., Brossart P., Cant C., Cella M., Colonna M., Brugger W., Kanz L., Ullrich A., Bühring H.J. (2001). Signal-regulatory protein alpha (SIRPalpha) but not SIRPbeta is involved in T-cell activation, binds to CD47 with high affinity, and is expressed on immature CD34+CD38− hematopoietic cells. Blood.

[76] Weng C.H., Assouvie A., Dong L., Beltra J.C., Budhu S., Mangarin L., Marouf Y., Morgado-Palacin L., Liu C., Monette S. (2025). Thrombospondin-1-CD47 signaling contributes to the development of T cell exhaustion in cancer. Nat. Immunol..

[77] Matthaios D., Tolia M., Mauri D., Kamposioras K., Karamouzis M. (2021). YAP/Hippo Pathway and Cancer Immunity: It Takes Two to Tango. Biomedicines.

[78] Cui H., Zhao G., Lu Y., Zuo S., Duan D., Luo X., Zhao H., Li J., Zeng Z., Chen Q. (2025). TIMER3: An enhanced resource for tumor immune analysis. Nucleic Acids Res..

[79] Schwartz A.L., Nath P.R., Allgauer M., Lessey-Morillon E.C., Sipes J.M., Ridnour L.A., Morillon Ii Y.M., Yu Z., Restifo N.P., Roberts D.D. (2019). Antisense targeting of CD47 enhances human cytotoxic T-cell activity and increases survival of mice bearing B16 melanoma when combined with anti-CTLA4 and tumor irradiation. Cancer Immunol. Immunother..

[80] Hu T., Liu H., Liang Z., Wang F., Zhou C., Zheng X., Zhang Y., Song Y., Hu J., He X. (2020). Tumor-intrinsic CD47 signal regulates glycolysis and promotes colorectal cancer cell growth and metastasis. Theranostics.

[81] Shklovskaya E., Lee J.H., Lim S.Y., Stewart A., Pedersen B., Ferguson P., Saw R.P., Thompson J.F., Shivalingam B., Carlino M.S. (2020). Tumor MHC Expression Guides First-Line Immunotherapy Selection in Melanoma. Cancers.

[82] Chen L., Zheng X., Huang H., Feng C., Wu S., Chen R., Jiang H., Yuan M., Fu Y., Ying H. (2023). Cordycepin synergizes with CTLA-4 blockade to remodel the tumor microenvironment for enhanced cancer immunotherapy. Int. Immunopharmacol..

[83] Eng C., Lakhani N.J., Philip P.A., Schneider C., Johnson B., Kardosh A., Chao M.P., Patnaik A., Shihadeh F., Lee Y. (2025). A Phase 1b/2 Study of the Anti-CD47 Antibody Magrolimab with Cetuximab in Patients with Colorectal Cancer and Other Solid Tumors. Target. Oncol..

[84] Jiang Z., Sun H., Yu J., Tian W., Song Y. (2021). Targeting CD47 for cancer immunotherapy. J. Hematol. Oncol..

[85] Ward-Hartstonge K.A., Kemp R.A. (2017). Regulatory T-cell heterogeneity and the cancer immune response. Clin. Transl. Immunol..

[86] Sokólska W., Gudowska-Sawczuk M., Orywal K. (2026). Cytokines and Chemokines as Emerging Biomarkers and Therapeutic Targets in Colorectal Cancer-Narrative Review. Int. J. Mol. Sci..

[87] Kim H.S., Nam J.S. (2025). The multifaceted role of YAP in the tumor microenvironment and its therapeutic implications in cancer. Exp. Mol. Med..

[88] Hu X., Manner K., DeJesus R., White K., Gattis C., Ngo P., Bandoro C., Tham E., Chu E.Y., Young C. (2023). Hypoimmune anti-CD19 chimeric antigen receptor T cells provide lasting tumor control in fully immunocompetent allogeneic humanized mice. Nat. Commun..

[89] Yamada-Hunter S.A., Theruvath J., McIntosh B.J., Freitas K.A., Lin F., Radosevich M.T., Leruste A., Dhingra S., Martinez-Velez N., Xu P. (2024). Engineered CD47 protects T cells for enhanced antitumour immunity. Nature.

